# Developing a panel of biomarkers and miRNA in patients with myocardial infarction for early intervention strategies of heart failure in West Virginian population

**DOI:** 10.1371/journal.pone.0205329

**Published:** 2018-10-24

**Authors:** Hari Vishal Lakhani, Tilak Khanal, Alaa Gabi, George Yousef, Mian Bilal Alam, Dana Sharma, Haytham Aljoudi, Nitin Puri, Ellen Thompson, Joseph I. Shapiro, Komal Sodhi

**Affiliations:** 1 Department of Internal Medicine, Marshall University Joan C Edwards School of Medicine, Huntington, WV, United States of America; 2 Division of Cardiology, Department of Internal Medicine, Marshall University Joan C Edwards School of Medicine, Huntington, WV, United States of America; 3 Departments of Biomedical Sciences, Marshall University Joan C Edwards School of Medicine, Huntington, WV, United States of America; 4 Departments of Surgery and Biomedical Sciences, Marshall University Joan C Edwards School of Medicine, Huntington, WV, United States of America; Universita degli Studi di Catania, ITALY

## Abstract

**Background:**

Myocardial infarction is the most common cause of heart failure. MI has been intricately linked to ventricular remodeling, subsequently leading to the reduction in the cardiac ejection fraction causing HF. The cumulative line of evidence suggests an important role of several biomarkers in modulating the cardiac vasculature, further contributing towards the progression of post-MI complications. Studies have demonstrated, yet not fully established, that an important biomarker, IL-10, has a causal relationship with MI and associated cardiac dysfunction.

**Hypothesis:**

This study aims to establish the role of IL-10 as a prognostic marker for the cardiovascular outcomes and to develop a panel of biomarkers and circulating miRNAs that could potentially result in the early detection of HF resulting from MI, allowing for early intervention strategies.

**Methods and results:**

Blood was withdrawn and echocardiography assessment was performed on a total of 43 patients that were enrolled, within 24 hours of the incidence of MI. Patients were divided in three main groups, based on the ejection fraction measurement from echocardiography: control (n = 14), MI with normal EF (MI+NEF, n = 13) and MI with low EF (MI+LEF, n = 16). Our results showed that TGFβ-1, TNF-α, IL-6 and MMP-9 were upregulated significantly in MI+NEF group and more so in MI+LEF group, as compared to control group (p<0.01). The circulating levels of miR-34a, miR-208b and miR-126 were positively correlated and showed elevated levels in the MI+NEF group, even higher in MI+LEF group, while levels of miR-24 and miR-29a were reduced in MI+NEF, and much lower in MI+LEF, as compared to the control group (p<0.01). Our results also demonstrated a direct correlation of IL-10 with the ejection fraction in patients with MI: IL-10 was elevated in MI+NEF group, however, the levels were significantly low in MI+LEF group suggesting an important role of IL-10 in predicting heart failure. Importantly, our study confirmed the correlation of IL-10 with EF by our follow-up echocardiography assessment that was performed 2 months after the incidence of MI.

**Conclusion:**

Our results support the clinical application of these serum biomarkers to develop a panel for appropriate prognosis and management of adverse cardiac remodeling and development of heart failure post-myocardial infarction.

## Introduction

Heart failure (HF) is common after acute myocardial infarction (MI), and has been associated with excess mortality. MI can lead to HF via several factors including ventricular remodeling, infarct size, and recurrent myocardial ischemia [1, 2]. The Framingham Heart Study, which examined the long term trends of HF after MI, found that HF post-MI occurred in 24.4% of the study population over a 30-year period. The study also found that there was an increase in the 30-day incidence of HF post-MI from 10% to 23.1% during the 30-year study. This may be attributed to an increased survival rate post-MI [3–5]. However, others have reported that 70% of patients who develop HF after an MI, on average, die within 7.6 a year period [6]. Thus, early detection and treatment are critical to improve morbidity and mortality outcomes.

In West Virginia (WV), utilizing a biomarker panel that detects HF post-MI is especially applicable due to the high prevalence of cardiovascular disease (CVD) and CVD risk factors in the state. WV has a population with one of the highest prevalence of diabetes at 12.0% and one of the highest rates of obesity in the United States with a rate of more than 35% [7, 8]. Given the prevalence of MI in WV is 6%, the highest of any state, and with most of the state having limited access to healthcare, it is necessary to formulate an alternate means to diagnose HF post-MI to optimize care [9].

Diagnosis of HF after MI is usually done with imaging in combination with laboratory testing. Typical methods can include chest radiography, but echocardiography is the most commonly used method for detecting the amount of ventricular dysfunction following an MI [10]. The cumulative line of evidence suggests that the measurement of ejection fraction (EF), based on the echocardiography assessment, is a strong determinant of HF and predictor of mortality [11–14]. EF determines the extent of volumetric fraction of blood that is pumped with each contraction. However, the reduction in the EF over the period of time is a strong indicator of progressive cardiac muscle damage, which may be caused by the blockage of coronary arteries limiting the blood flow, impaired mitral valve function, irregular heart rhythm and the narrowing of the aortic valves. Once detected, treatment methods for HF patients’ post-MI vary. This can include ace inhibitors, angiotensin-II receptor blockers, β-blockers, aldosterone antagonists, lipid lower therapy, implantable cardioverter defibrillators, and potentially stem cell therapy [2, 15–17]. These forms of treatment have variable success depending on when HF is detected and treatment initiated.

It would be beneficial if HF post-MI could be detected and attenuated prior to irreversible complications or death. This could be accomplished through the creation of a biomarker panel to detect HF post-MI. Traditional serum biomarkers are valuable because they allow for earlier diagnosis of disease and can be measured with relative ease [18]. MicroRNAs are valuable as biomarkers because they are stable in various body fluids, their levels can be assessed by various methods, the sequences of most miRNAs are conserved among different species, and the expression of some miRNAs are specific to individual tissues or biological states [19].

Based on a review of the literature, we are proposing a biomarker panel to detect HF post-MI with the primary focus on interleukin 10 (IL-10). IL-10 is a multifunctional cytokine with a large role in the limitation and termination of inflammatory responses [20]. It has been demonstrated that IL-10 levels play a significant role in the development of HF post-MI. In addition to IL-10, we are proposing other biomarkers including transforming growth factor beta (TGFβ), matrix metalloproteinase-9 (MMP-9), tumor necrosis factor (TNF α), and interleukin 6 (IL-6), which are crucial in the fibrotic and inflammatory cardiovascular damage. We further assessed the circulating levels of several miRNAs including, miRNA-24, miRNA-29a, miRNA-34a, miRNA-208b, and miRNA-126, that plays a critical role in stimulating cardiac remodeling, cardiac vasculature damage and contribute in the underlying pathological mechanisms involved in HF. Based on this biomarker panel, detection of HF post-MI would allow for earlier attenuation of disease leading to a decrease in the mortality rate, an increase in successful treatment outcomes, and a decrease in overall healthcare costs.

## Material and method

### Patients

A total of 43 adult patients, visiting the Cardiology Clinic at Marshall University School of Medicine, were recruited for this study. Each patient was briefed about the use of the blood sample for this clinical study and each patient signed an informed consent. The patients were divided in three main groups based on their incidence of MI and the percentage EF: patients with no incidence of MI and normal EF, (Control, n = 14); patients with a clinical diagnosis of MI but with normal EF (MI+NEF, n = 13); patients with an incidence of MI and low EF, (MI+LEF, n = 16). Patients with any hematologic disorder, trauma, autoimmune disease, acute or chronic liver or kidney disease and any form of cancer were excluded from the study. The patients’ follow-up protocol was maintained and echocardiography was performed after 2 months of the initial diagnosis/symptoms of MI for the assessment of cardiac function. To ensure an appropriate selection of patients eligible for the study, trained hospital personnel examined patients’ medical records with appropriate confidentiality measures and in compliance with HIPAA. The Ethics Committee of the Cabell Huntington Hospital, West Virginia approved the study.

### Blood samples

Standard protocol was followed by trained personnel to withdraw venous blood from the eligible patients within 24 hours of the incidence of MI. A total of approximately 5 mL of blood was withdrawn from antecubital vein into the BD Vacutainer for the analysis of the biomarkers and evaluation of expression levels of miRNAs in the samples. The blood was centrifuged within 30 minutes of withdrawal at 10,000 rpm for 10 minutes under temperature settings of 4˚C. The serum was separated from the blood after centrifugation and collected in appropriately labeled Eppendorf tubes. The serum was further distributed to make aliquots of each sample to prevent continuous freeze-thaw cycle. All of the samples were stored under -80 C to be used further for quantification of biomarkers and miRNAs.

### Analysis of biomarkers

Enzyme-Linked Immunosorbent Assay (ELISA) was done for the quantification of the plasma biomarkers. The manufacturer’s protocol was followed for each of the following ELISA kit: IL-6 (Abcam), TGFβ-1 (R&D Systems), MMP-9 (Abcam), TNF-α (Abcam), and IL-10 (Sigma Aldrich). The assay was performed in a 96-well plate and at the end of protocol; the plate was read at 450nm wavelength, in BioTek ELx800 Absorbance Reader. A standard curve graph was plotted for each biomarker and the concentrations were calculated using the resulting equation from the line of best fit.

### Quantification of miRNA

RNA Extraction was done using miRNeasy Serum Plasma Kit (Qiagen, Hilden, Germany). We followed manufacturer’s protocol to extract RNA from our serum samples and further analyzed the quantity and quality of our RNA by 260:280 ratio using NanoDrop Analyzer (Thermo Scientific). Following the RNA extraction, we used miRCURY LNA Universal RT microRNA PCR Kit (Exiqon, Vedbaek, Denmark) for our RT reactions, to prepare cDNA, with 50ng of total RNA for each reaction. Further, miRNA specific primers were used, combined with SYBR green master mix, to perform RT-PCR reaction. Three technical replicates were used for each sample allowing more accuracy in the final qRT-PCR amplification data which was run on a 7500 Fast Real Time PCR System (Applied Biosystems). This protocol has been detailed previously [21]. Following is the sequence of miRNAs:

hsa-miR-24-3p (UGGCUCAGUUCAGCAGGAACAG);

hsa-miR-34a-5p (UGGCAGUGUCUUAGCUGGUUGU);

hsa-miR-29a-3p (UAGCACCAUCUGAAAUCGGUUA);

hsa-miR-208b-3p (AUAAGACGAACAAAAGGUUUGU);

hsa-miR-126 (UCGUACCGUGAGUAAUAAUGCG).

### Echocardiography

2D Doppler and color flow imaging was used to perform transthoracic echocardiography on patients. A certified echo technician performed the study on the patients using Philips IE 33 with a S5 transducer in an ICAEL- accredited laboratory. The activity was performed under the set guidelines by the American Society of Echocardiography. Further, 2D imaging allowed for the evaluation of EF which was calculated as detailed previously.

## Results

### Patients’ demographics and characteristics associated with MI, compared to healthy control population

The patients’ clinical profile provided a deeper insight to cardiovascular risk factors in each group. Demographic and clinical characteristics of the entire patient population, based on each group, are depicted in Table 1. All patients were adults’ including 26 males and 17 females, however, there was no significant difference among the groups in terms of age. Overall, significantly greater number of patients in MI+NEF group and MI+LEF group had hypertension, diabetes mellitus, and hypercholesterolemia and were positive for smoking status, as opposed to the control group. The fasting blood glucose values were also retrieved for each patient from their clinical profiles, with the mean values for MI+NEF and MI+LEF, suggesting a positive trend towards diabetes as compared to the control group. Troponin I, a marker for ischemic damage, was significantly elevated in MI+NEF and MI+LEF group as compared to control group (p<0.01) that had a normal cardiac function. There was also a significant difference in the lipid profile of each group, showing elevated triglyceride levels and reduced high-density lipid (HDL) values in MI+NEF and MI+LEF groups, as compared to the control group (p<0.01).

**Table 1:** Patient demographics and general clinical profile.

Groups	Control	MI+NEF	MI+LEF
Sample Size (n)	14	13	16
Age (yrs.)	53.2 ± 1.9	64.4 ± 2.5	59.7 ± 2.1
Sex (M/F)	7/7	9/4	10/6
**Cardiovascular Risk Factors**			
Smoking Status (%)	3 (21.4)	9 (69.2)	7 (43.8)
Diabetes Mellitus (%)	2 (14.3)	8 (61.5)	5 (31.2)
Hypertension (%)	2 (14.3)	9 (69.2)	9 (56.2)
Hypercholesterolemia (%)	2 (14.3)	10 (76.9)	7 (43.8)
Fasting Blood Glucose (mg/dL)	91.0 ± 4.2	120 ± 9.8	135.5 ± 10.8 **
Blood Pressure (SBP/DBP)	116 / 78	142 / 81	132 / 80
Triglyceride (mg/dL)	57.08 ± 8.3	152.08 ± 12.4**	114.6 ± 18.0**
High Density Lipid (HDL, mg/dL)	63.3 ± 5.2	44.6 ± 4.7**	40.1 ± 2.7**
Low Density Lipid (LDL, mg/dL)	72.8 ± 2.9	100.3 ± 7.3	103.6 ± 10.6
Troponin	-	18.23 ± 5.0**	29.5 ± 3.9**

Characteristics of the patient population recruited for the study, demonstrating basic clinical profile to assess cardiovascular risk factors. Values represent means ± SEM. Patient groups: Control (n = 14), MI+NEF (n = 13), and MI+LEF (n = 16).

*p<0.05 vs. Control

**p<0.01 vs. Control.

### Evaluation of plasma biomarkers in patients with MI compared to healthy control population

**Fig 1:**
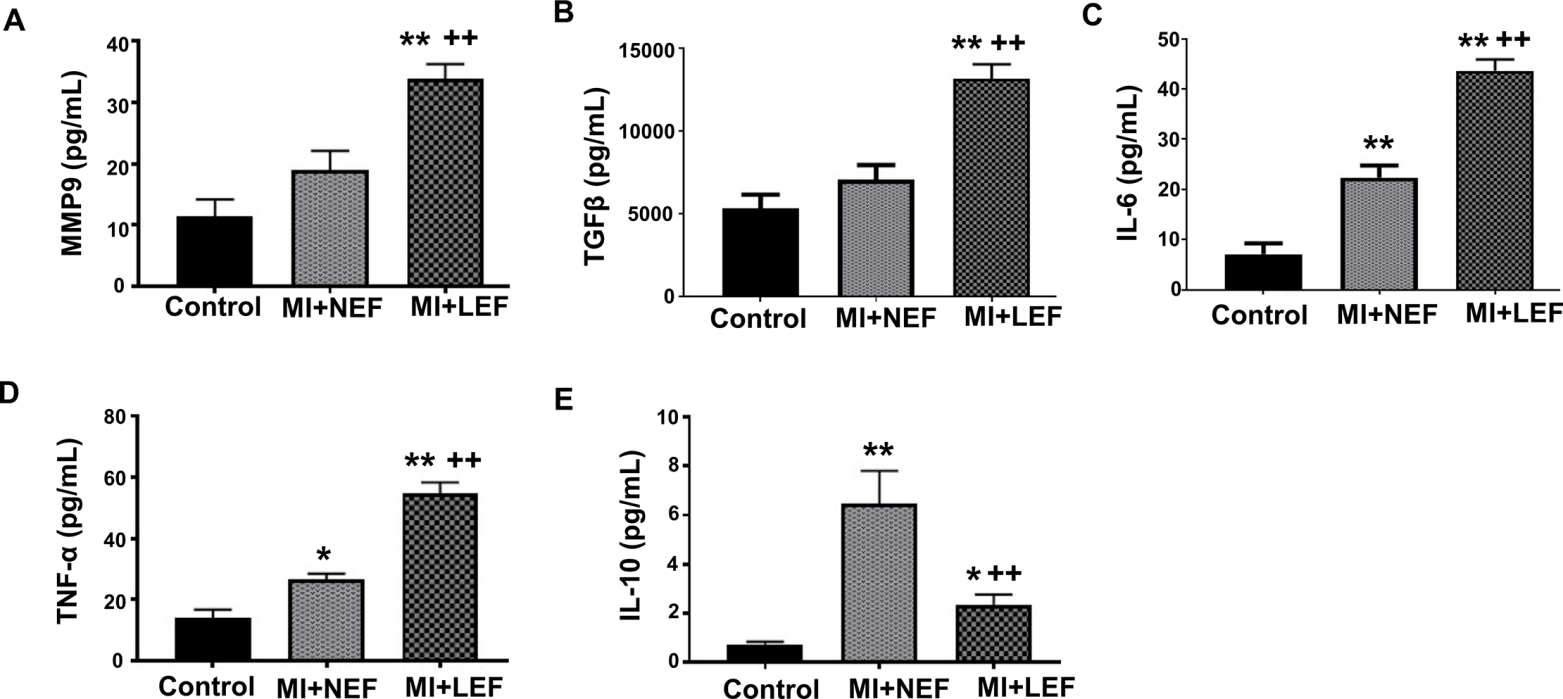
Quantitative analysis by ELISA for plasma concentrations of (A) MMP9, (B) TGFβ, (C) IL-10, (D) TNF-α and (E) IL-6. Values represent means ± SEM. *p<0.05 vs. Control, **p<0.01 vs. Control, ^++^p<0.01 vs. MI+NEF. Patient groups: Control (n = 14), MI+NEF (n = 13), and MI+LEF (n = 16).

Blood plasma was collected from patients within 24 hours of incidence of MI and the concentration of biomarkers was evaluated in each group, as shown in Fig 1. The levels of a key marker for cardiac remodeling, MMP-9, and the concentration of polypeptide protein, TGFβ-1 was noted to be significantly elevated in MI+LEF patients, in comparison with the control and MI+NEF group (p<0.01), as shown in Fig 1A and Fig 1B. The pro-inflammatory cytokines IL-6 and TNF-α was also noted to be significantly higher in MI+NEF group and further increased in MI+LEF group, as compared to patients’ in control group (Fig 1C and Fig 1D). IL-10 is multi-functional, immuno-regulatory, and anti-inflammatory cytokine that tends to balance the pro-inflammatory insults. Our study group of MI+NEF had significant increase in the levels of IL-10 as compared to control. Interestingly, our results showed reduced level of IL-10 in MI+LEF group, as compared to patients in MI+NEF and control group (Fig 1E).

### Evaluation of circulating miRNA Biomarkers in Patients with MI compared to Healthy Control Population

**Fig 2:**
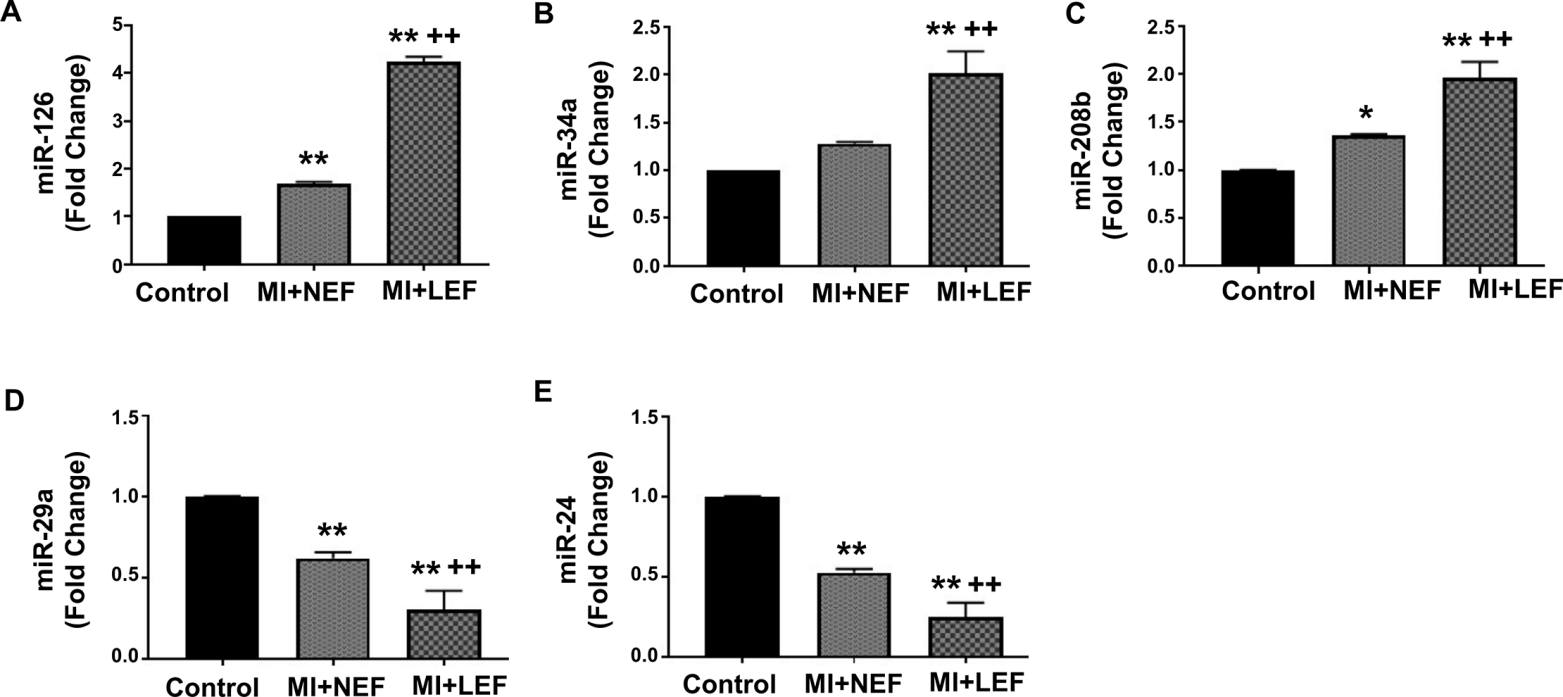
qRT-PCR Analysis of circulating level of miRNAs (A) miR-24, (B) miR-29a, (C) miR-34a, (D) miR-126 and (E) miR-208b. Values represent means ± SEM. *p<0.05 vs. Control, **p<0.01 vs. Control, ^++^p<0.01 vs. MI+NEF. Patient groups: Control (n = 14), MI+NEF (n = 13) and MI+LEF (n = 16).

The miRNAs play a vital role in the regulation of genetic expression and cardiac remodeling, but altered levels of miRNAs may result in deleterious effects and aggravation of cardiac damage. Our result shows significant evidence for a correlation of miRNAs with the MI groups having normal or low EF. The circulating levels of miR-126, miR-34a and 208b were elevated in the setting of MI+NEF, however, the levels for these miRNAs showed greatest increase in the MI+LEF group as compared to control and MI+NEF (Fig 2A, Fig 2B and Fig 2C). On the other hand, the levels of miR-29a and miR-24 were significantly reduced in the MI+NEF group, as compared to control, while demonstrated further decrease in the MI+LEF group, in comparison to control and MI+NEF group (Fig 2D and Fig 2E).

### Echocardiography assessment in patients with MI to evaluate left ventricular EF

**Fig 3:**
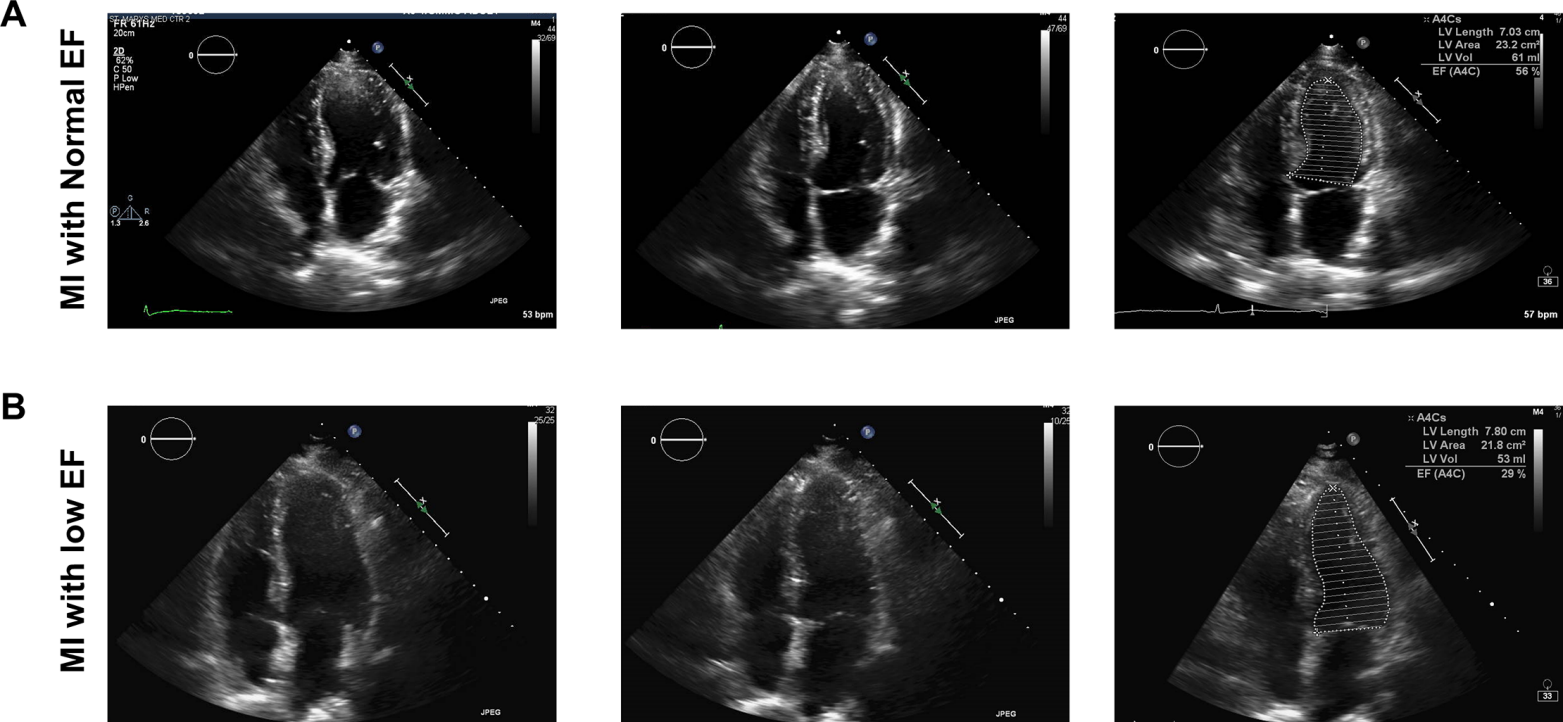
Representative transthoracic echocardiographic images from post-MI patients performed within 48 hours of incidence of MI, to evaluate EF. Images were assessed using biplane method in end diastole and end systole respectively. (A) Evaluation of cardiac function in patients with (A) MI with normal EF and (B) MI with low EF.

All patients underwent transthoracic echocardiography post-MI within 48 hours. Patients were evaluated for EF, comparing systole and diastole activity through this imaging activity. Fig 3 illustrates representative echocardiographic image of the left ventricle from the apical 4-chamber view and shows a 2D frame at left ventricular diastole and systole, for MI patients with normal or low EF. Fig 3A demonstrates left ventricular function in a MI patient with normal EF (56%). Fig 3B shows a less inward motion of the left ventricular walls in patients with low EF (29%), as compared to normal EF. These echocardiographic images use Simpson’s biplane method in end diastole and end systole respectively to calculate EF.

### Co-relation of IL-10 levels and EF in patients with post-MI

**Fig 4:**
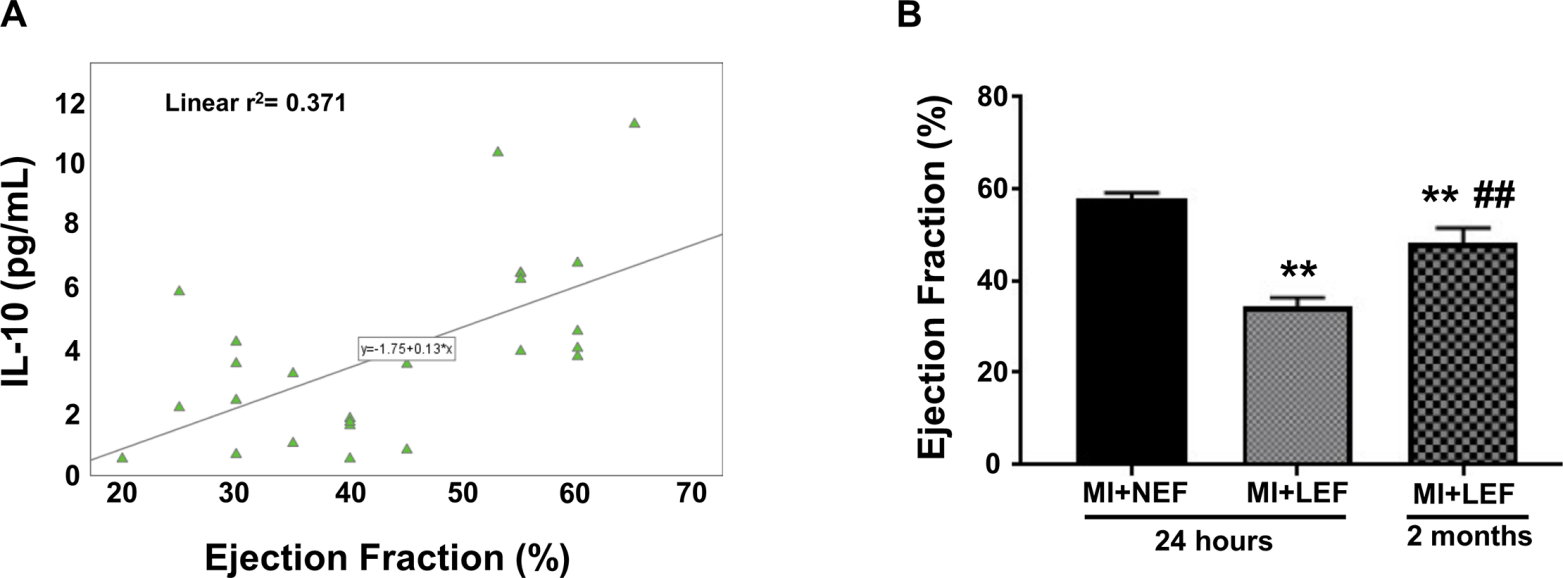
Association of IL-10 and EF in post-MI patients. (A) Statistical analysis performed using SPSS software to examine correlation between EF and IL-10 estimating Spearman’s correlation coefficient. (B) Comparison of EF after 2month follow-up in patients with MI with low IF. Values represent means ± SEM. **p<0.01 vs. MI+NEF, ^##^p<0.01 vs. MI+LEF.

We examined the co-relation between cytokine IL-10 and EF in patients with post-MI. Statistical analysis was performed using SPSS software and correlation between EF and IL-10 was examined by estimating Spearman’s correlation coefficient. Our results showed a direct positive correlation between the IL-10 levels and EF, demonstrating that low EF is associated with reduced IL-10 levels in post-MI patients, as shown in Fig 4A. Patients with low EF were also re-assessed, as they underwent echocardiography in their follow-up after 2 months of the incidence of MI. A focused analysis was performed in the subset of MI participants with low EF, with the initiation of therapeutic interventions, which included ace-inhibitors and β-blockers, to compare EF at 2-month follow-up. Our results showed significantly low EF, even after 2 months of pharmacological interventions, as compared to the MI+NEF group (p<0.01) (Fig 4B). As expected, the EF was improved after 2 months as compared to our 24-hour EF evaluation in MI+LEF group (p<0.01).

## Discussion

**Fig 5:**
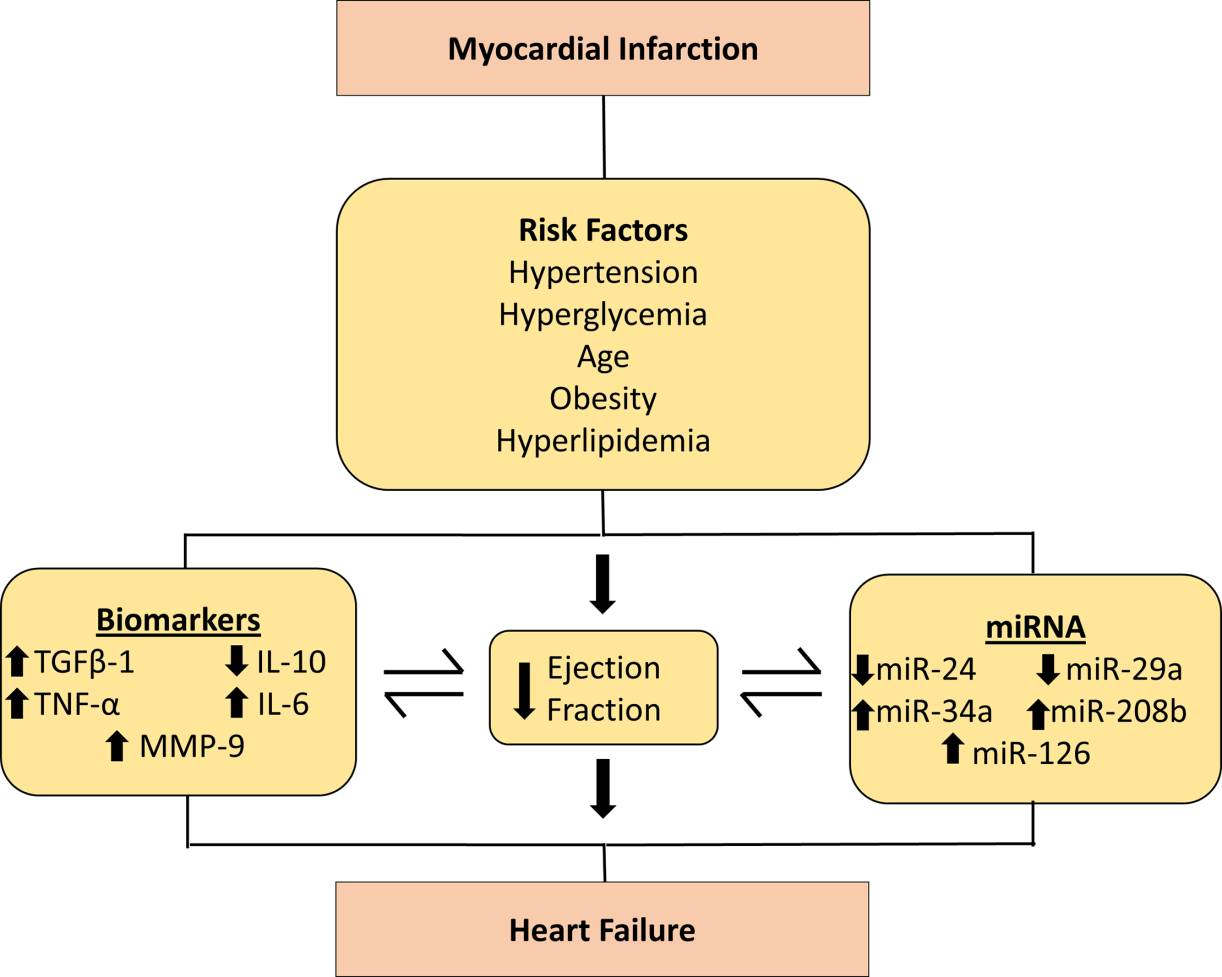
Schematic representation illustrating the association of EF with serum biomarkers and expression level of circulating miRNAs in the progression to HF post-MI.

MI is characterized by the acute degradation of the myocardial integrity, which is often augmented by the ventricular damage and progressive degeneration and decline in cardiac function [22]. Clinical evidence suggests that the intrinsic cardiac damage makes the heart more vulnerable to stress and contributes to an intense inflammatory response, leading to an accelerated cardiovascular morbidity and mortality in the patients with an incidence of MI [23]. Despite several diagnostic approaches that have demonstrated a great pharmacological impact, the current strategies are not cost effective, since most of the patients require frequent echocardiographs [24]. In concordance, the echocardiograph assessment lacks sensitivity and specificity in the early prediction of the development of cardiac dysfunction. This implies that echocardiograph is a reliable tool only for the demonstration and identification of the cardiac damage in the patients that already has cardiac dysfunction, which suggests a need for the prognostic tools that can prove to be essential in the early screening [25]. This study evaluated novel panel of biomarkers and circulating miRNAs to detect early stage HF patients post MI event, which will enable attenuation of disease progression prior to the onset of irreversible complications in West Virginian population. The results of the present study indicate the co-relation of plasma IL-10 levels with the EF in the first 24 hours of MI event in patients, that further progresses to HF. Although, the IL-10 levels were only determined at the first incidence of MI, the results provide significant evidence suggesting their importance as a potential prognostic marker in such patients, in order to develop early intervention strategies. The overall results are summarized in a schematic diagram (Fig 5) demonstrating all potential markers and their significance in early detection and determining the prognosis of HF in West Virginian population.

Despite the advances in intrusive and pharmacological treatment of MI, long-term prognosis is still hampered by the risk of HF in spite of post-infarction cardiovascular remodeling. The evaluation of clinical parameters and cardiovascular risk factors is essential in the determination of cardiovascular dysfunction. These risk factors have been known to lead to the development and progression of MI, however, prolonged exposure to such cardiovascular risks can pre-dominantly influence complications associated with MI, such as HF. Such risk factor includes, age, smoking status, diabetes, hypertension, and hypercholesterolemia. Similarly, assessment of other quantitative variables is essential in pre- and post-MI conditions which primarily includes, plasma glucose levels, body mass index (BMI), high density lipid (HDL), low density lipid (LDL) and triglycerides levels. These factors are considered instrumental in advocating the deleterious effects on the heart, further weakening its overall function and attributing atherosclerotic effects. Important MI marker, Troponin I, an inhibitory protein present in the myocardium, is a crucial diagnostic marker for MI, which regulates the muscle function of the heart [26]. The slightest elevation of this protein level from the normal range indicates an ischemic damage making this as one of the important characteristic of the onset of MI [26]. These characteristics are summarized in Table 1, and are crucial diagnostic markers that may be monitored post-MI in order to attenuate progression to HF.

This study stipulates the importance of IL-10 in the myocardial insults observed post-MI. IL-10 is a potent anti-inflammatory cytokine that mediates the persistent inflammatory response by suppressing the expression of inflammatory cytokines [23]. However, when inflammatory response exceeds the detoxifying ability of the cells, the adverse effects are followed by impaired left ventricular function and remodeling [27–29]. IL-10 has also been demonstrated to have anti-atherosclerotic properties by downregulation of cardiac adhesion molecules [30, 31], production of lytic enzymes and stabilizing atheromatous plaques [32]. Based on the literature review, there are no known studies that demonstrate diabetes and metabolic syndrome influencing levels of IL-10 and modulating outcomes in MI. However, further studies might need to be conducted in order to scientifically establish a potential relationship among the metabolic parameters, IL-10 and MI. In concordance with the mechanistic action of IL-10, our results demonstrate that the reduced levels of IL-10 are directly correlated with the EF, suggesting that the low IL-10 levels can propagate HF and can prove to be an important predictive marker for the early prognosis of HF. The heightened levels of IL-10 in MI+NEF group may contemplate a counter-regulatory mechanism in the cardiac state where the levels of pro-inflammatory cytokines are significantly elevated. Numerous studies have substantiated the role of IL-10 in their in vivo models, demonstrating that the treatment with IL-10 attenuates MI-induced cardiac cell death, left ventricular fibrosis and the suppression of inflammatory pathways including p38 mitogen activated protein (MAP) kinase [23]. However, there have been some limitations due to the contradictory evidence present in the literature. The observed discrepancies were result of possible variations in the collection time of blood plasma to evaluate IL-10 levels, while it may also be a ramification of multiple genetic variations that may lead to different outcomes [33, 34]. The association of IL-10 with the percentage of cardiac EF in patients with MI may ratify these discrepancies and corroborate IL-10 to be an important predictive marker of cardiovascular outcome in patients with MI incidence.

Our study further set out the importance of a panel of biomarkers and circulating levels of miRNA, in addition to IL-10; these markers play an important role in the early detection of HF and cardiac fibrosis, enabling early intervention to attenuate disease progression. Our results provide significant evidence that altered expression levels of biomarkers potentially attribute to HF. An important biomarker, a polypeptide protein, TGFβ-1, was also elevated in MI+NEF group and demonstrated further increase in MI+LEF group. The inclination of this biomarker is associated with its distinct role in ventricular remodeling and alteration of myofibroblastic phenotype [35]. TGFβ-1 promotes the fibrous deposition in the cardiac tissues by inducing the synthesis of collagen [36] and reduction in the expression of collagenase [37], also contributing towards cardiomyocytes hypertrophy [38] eventually leading to the cardiac damage. Similarly, the change in the levels of pro-inflammatory cytokine, TNF-α, suggests its important role in the pathogenesis of HF, which has been reported in other studies. Multiple studies suggest that the production of TNF-α is stimulated in case of cardiac injury that further promotes inflammatory state in cardiomyocytes [39, 40]. This cytokine mediates several pathophysiological processes and causes inflammatory response in cardiac shock further assisting in progression of HF. Several studies have noted to perform anti-TNF-α therapy to attenuate the progression to HF in order to reverse the inflammatory damage [39]. Our result showed TNF-α was significantly higher in MI+NEF group and even further elevated in MI+LEF group, as compared to the healthy control patients. The cytokine, IL-6, also tends to play an important role in the inflammatory process post-MI with complex pathological process which has been studied before. IL-6 is suggested to be a part of formation of atherosclerotic plaque and ventricular dysfunction [41]. Our results were in concordance with the previous findings and showed the elevated levels in MI patients with normal or low EF that correlates with its inflammatory mechanism and myocardial necrosis. Subsequently, a biomarker from a comparatively novel class of collagenolytic enzyme, MMP9, that promotes the degeneration of extracellular matrix also showed elevated levels in MI patients which suggest its key role in the progression to HF. Several findings have shown that the levels of MMP-9 are increased after MI, due to its role as a mediator of cardiac remodeling, making it as an independent risk factor of HF post MI. Subsequent with the previous studies, it is assumed that the MMP-9 aggravates ischemia and facilitates the infiltration of neutrophils, also causing extracellular matrix degradation due to its unique characteristic from matrix enzyme family [42].

Based on the literature review and their specific role in modulating cardiovascular function and damage, circulating levels of several miRNAs were included in the study. Evidence suggests that mechanistically, the induction or repression of miRNA can trigger cell type specific downstream signaling cascades and the expression of miRNA plays a crucial role in the regulation of overall cardiac function [43]. Furthermore, several studies have reported a critical role of miRNAs-dependent mechanisms in the regulation of cardiac angiogenesis, fibrosis and cardiomyocyte hypertrophy in an incidence of MI [43–45]. While the underlying molecular mechanisms are not yet fully understood, the pathways associated with miRNA biogenesis have been substantially considered important for overall cardiac function. Due to the novelty of miRNAs and their potential mechanisms, there has been a controversial evidence suggesting that the higher levels of miRNAs are due to the cellular death which causes the release of miRNAs in circulation, while the lower circulating levels of miRNAs might suggest a possible cellular uptake from the circulation that restores the impaired intracellular mechanisms that leads to the development and progression to HF [46]. In our study, the circulating levels of these miRNAs were significantly correlated in MI patients when compared to the control group that indicates the importance of their inclusion in this panel of biomarkers while understanding their individual role. Expression of miR-24 was downregulated in MI patients due to its involvement in the dynamic process of cardiac fibrosis after MI [44]. MiR-24 is also found to regulate the activity of TGFβ-1 and along with this marker, it could potentially mediate development of cardiovascular structure post-MI [44]. The downregulation of miR-24 could be an indicator of potential progression to HF, as shown in our results demonstrating reduced levels of miR-24 in MI+NEF group and further reduction in MI+LEF group as compared to control. Similarly, miR-29a was reduced in our MI study group due to its essential role in cardiac remodeling after MI. Evidence suggests that the downregulation of miR-29a was persistent in cardiac fibrosis tissue by the regulation of vascular endothelial growth factor-A (VEGF-A) and mitogen activated protein kinase (MAPK) signaling pathway, causing suppression of cardiac fibroblasts proliferation [47]. Furthermore, miR-29a has also been associated with the targeting of extracellular matrix genes, including collagen and MMPs, with their expression in cardiomyocytes and the early phase of cardiac hypertrophy [48]. Other studies depict the elevated levels of miR-34a and miR-208b, which accounts for similar function in the process of cardiac remodeling in post MI patients. The role of miR-34a has been found to be as a stimulator of apoptotic factor in cardiac contractile function after MI, while miR-208b promotes the left ventricular dysfunction [49]. These biomarkers are remarkable in the progression to HF after an incidence of MI. Our results were indicative of elevated circulating levels of both miR-208b and miR-34a that were in concordance with the previous studies. Pharmacological strategies can be developed to silence these miRNAs in MI patients to attenuate the risk of HF. The circulating level of miR-126 was also elevated as it has been noted to regulate cardiovascular pathological processes [50]. The results of the circulating levels of miRNA biomarkers are illustrated in the schematic diagram (Fig 5), which shows an association in MI patients and can possibly prove to be predictor markers for the progression to HF. While there have been limited number of studies that elucidate the sex-based differences of these specific miRNAs in patients with MI and subsequent HF, several studies have demonstrated sexual dimorphism in various pathophysiological conditions, including ischemic cardiomyopathy, schizophrenia, metabolic diseases and liver fibrosis [51–54]. Nevertheless, it would be interesting to evaluate the outcomes of these miRNAs in patients with MI, and their subsequent impact on HF, by conducting an extensive future study based on gender profiling and the validation of these circulating miRNAs.

Conclusively, this novel panel of biomarkers can potentially allow early detection and regulation of cardiovascular function post MI in the West Virginian population. Along with the clinical parameters and biochemical markers, progression to HF post-MI can be attenuated and therapeutic measures can be taken by clinicians to abate the morbidity and mortality from HF which poses an economic and social burden on West Virginian population. Although further research is necessary to determine the predictive value of these tested markers, based on these results we have demonstrated that our formulated biomarker panel has significant potential in early detection, prognosis and management of the onset of HF post-MI. This biomarker panel could decrease the disease burden on our WV population by allowing for the earlier attenuation of disease prior to serious complications.

## Conclusion

HF post-MI places a major disease burden on the WV population. Our results demonstrated a dysregulation in all biomarkers including circulating levels of miRNAs, tested in patients with an incidence of MI having normal or low EF, as compared to the healthy control group. We have also observed changes in the EF of post-MI patients, as observed via echocardiography, which were associated with the levels of IL-10. Although further research is necessary to determine the predictive value of these tested markers, based on these results we have demonstrated that our formulated biomarker panel has significant potential in early detection, prognosis and management of the onset of HF post-MI, before the occurrence of irreversible complications. This biomarker panel could decrease the disease burden on our WV population by allowing for the earlier attenuation of disease prior to serious complications.

## Perspective

Our formulated biomarker panel has the potential for significant application in a clinical setting. Allowing for the early detection of the onset of HF post-MI, our biomarker panel would allow healthcare providers to begin attenuating the disease prior to the onset of irreversible complications. As a result, this would lead to a decreased mortality rate by increasing the number of successful treatment outcomes while also decreasing the burden of healthcare costs on the state of WV.

## Abbreviations

MI: Myocardial Infarction
HF: Heart Failure
EF: ejection fraction
NEF: normal ejection fraction
LEF: low ejection fraction
IL-10: Interleukin-10
TNF-α: tumor necrosis factor alpha
IL-6: Interleukin-6
MMP-9: matrix metallopeptidase 9
TGFβ-1: transforming growth factor beta 1
WV: West Virginia
BMI: body mass index
HDL: high density lipid
LDL: low density lipid
